# Synthesis, mol­ecular and crystal structure of 1-(1,2-di­hydro­phthalazin-1-yl­idene)-2-[1-(thio­phen-2-yl)ethyl­idene]hydrazine

**DOI:** 10.1107/S2056989019000732

**Published:** 2019-01-22

**Authors:** Felicite Majoumo-Mbe, Emmanuel Ngwang Nfor, Patrice Kenfack Tsobnang, Valoise Brenda Nguepmeni Eloundou, Joseph Ngwain Yong, Ikome Iris Efeti

**Affiliations:** a Department of Chemistry, University of Buea, PO Box 63 Buea, Cameroon; b Department of Chemistry, University of Dschang, PO Box 67, Dschang, Cameroon

**Keywords:** 2-Acetyl­thio­phene-1-phthalazinylhydrazone, bifurcated stacking inter­actions, tetra­mers, condensation reaction, co-planar rings, chevron-shaped motifs, C—H⋯π inter­actions, crystal structure

## Abstract

The title compound, C_14_H_12_N_4_S, was synthesized by the condensation reaction of hydralazine and 2-acetyl­thio­phene and during the reaction, a proton transfer from the imino nitro­gen atom to one of the endocylic nitro­gen atoms occurred. The compound crystallizes in the monoclinic crystal system with two independent mol­ecules (mol­ecules 1 and 2) in the asymmetric unit. In each mol­ecule, there is a slight difference in the orientation of the thio­phene ring with respect to phthalazine ring system, mol­ecule 1 showing a dihedral angle of 42.51 (1)° compared to 8.48 (1)° in mol­ecule 2. This implies an r.m.s deviation of 0.428 (1) Å between the two mol­ecules for the 19 non-H atoms. The two independent mol­ecules are connected *via* two N—H⋯N hydrogen bonds, forming dimers which inter­act by two bifurcated π–π stacking inter­actions to build tetra­meric motifs. The latter are packed in the *ac* plane *via* weak C—H⋯π inter­actions and along the *b* axis *via* C—H ⋯N and C—H⋯π inter­actions. This results a three-dimensional architecture with a tilted herringbone packing mode.

## Abstract 2

The two independent mol­ecules in the asymmetric unit of the title compound are connected *via* two N—H⋯N hydrogen bonds, forming dimers which inter­act by two bifurcated π–π stacking inter­actions to build tetra­meric motifs. These are packed *via* C—H ⋯N and C—H⋯π inter­actions, resulting in a three-dimensional architecture with a tilted herringbone packing mode.

## Chemical context   

Hydralazine compounds are being studied intensively for their biological and chemical properties, the former giving them inter­esting pharmacological properties (anti­microbial, anti­malarial and anti­tumor activity; Jackson *et al.*, 1990 ▸; Zelenin *et al.*, 1992 ▸; Kaminskas *et al.*, 2004 ▸; Vicini *et al.*, 2006 ▸). They also find wide applications in the treatment of tuberculosis, leprosy and mental disorder. Furthermore, there is considerable research inter­est in 1-hydrazinophthalazine (hydralazine) because its hydro­chloride is an effective drug for the emergency reduction of blood pressure in hypertensive crises (Draey & Tripod, 1967 ▸). It has also been reported that a combination of hydralazine and hydro­chloro­thia­zide is being used to treat high blood pressure, as they work by relaxing blood vessels and increasing the supply of blood oxygen to the heart while reducing its workload (Shoukry & Shoukry, 2008 ▸). The chemical properties of hydrazone compounds are also inter­esting because their nature as polydentate ligands makes them very versatile mol­ecules. The physiological importance of hydralazine derivatives has led to great inter­est in their complexation tendency with metal ions, especially with transition-metal ions of biological importance. The coordination chemistry of hydrazones is being studied in connection with their increasing use as pharmaceuticals and analytical reagents. Few complexes of 1-phthalazinylhydrazone have been reported (Al’-Assar *et al.*, 1992 ▸; Kogan *et al.*, 2009 ▸; Holló *et al.*, 2014 ▸; Bakale *et al.*, 2014*b* ▸; Levchenkov *et al.*, 2015 ▸). In a continuation of our studies of hydralazine derivatives and their complexes (Nfor *et al.*, 2013 ▸; Majoumo-Mbe *et al.*, 2015 ▸), we herein report the preparation and the structural study of the title compound, also known as 2-acetyl­thio­phene-1-phthalazinylhydrazone.
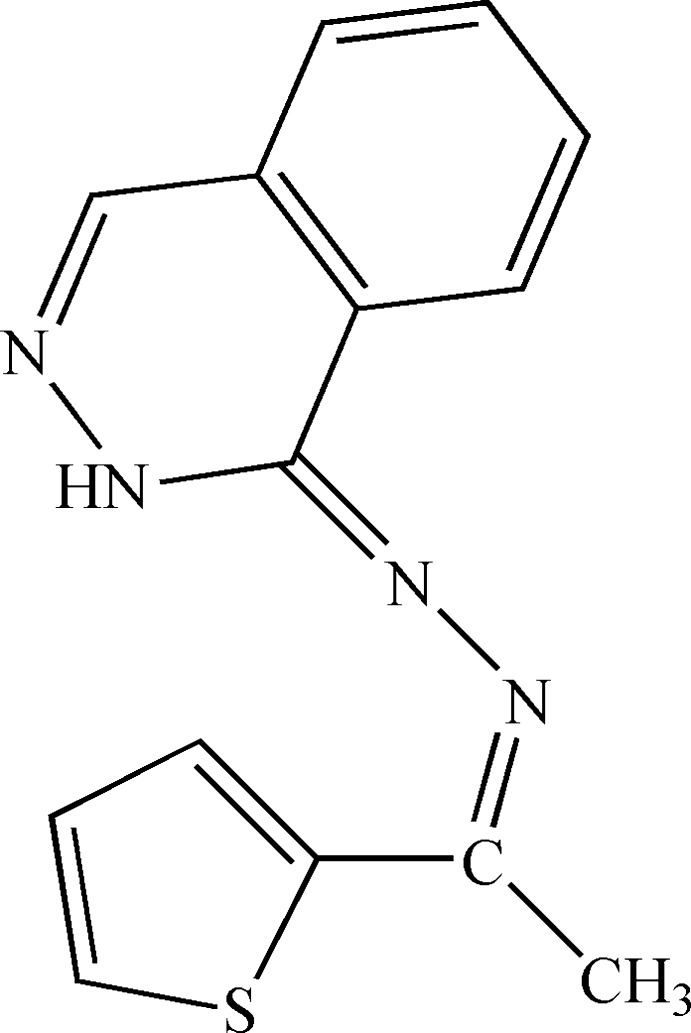


## Structural commentary   

**Figure 1:**
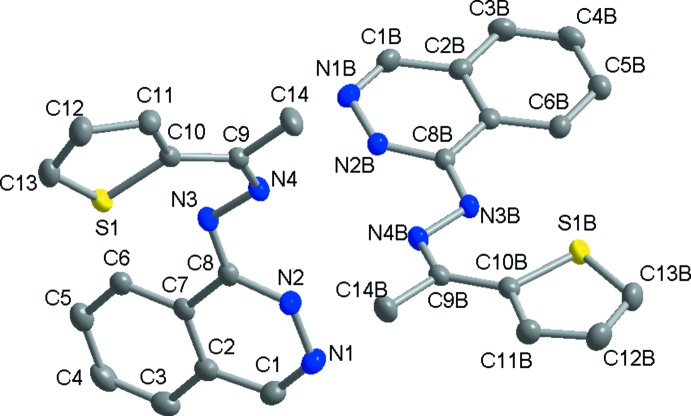
Mol­ecular structure of the two independent mol­ecules (1 and 2) with the labelling scheme. Displacement ellipsoids are drawn at the 50% probability level. H atoms have been omitted for clarity.

The title compound crystallizes in the monoclinic crystal system (space group *P*2_1_/*n*) with two independent mol­ecules, 1 and 2, in the asymmetric unit, as shown in Fig. 1 ▸ (atoms in mol­ecule 2 have the suffix *B*).

**Figure 2:**
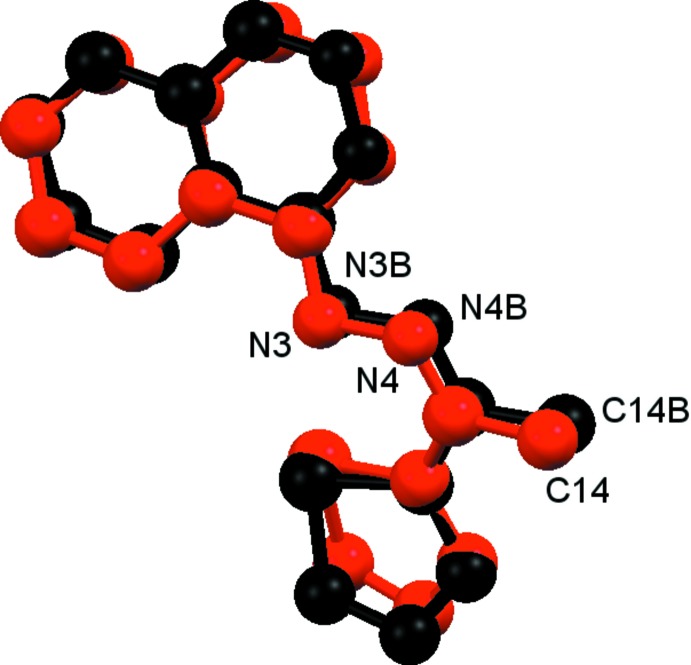
A view of the overlay (*Mercury*; Macrae *et al.*, 2006 ▸) of the two independent mol­ecules (colour code: red = mol­ecule 1, black = mol­ecule 2).

There are slight differences between the mol­ecules, as shown in Fig. 2 ▸, with an r.m.s. fit of 0.428 (1) Å for the 19 non-H atoms. This deviation arises from the different orientations of the thio­phene moiety. The dihedral angle between the thio­phene ring and the phthalazine ring system is 42.51 (1)° in mol­ecule 1 compared to 8.48 (1)° in mol­ecule 2.

In both mol­ecules, the thio­phene rings (C10–C13/S1) are in a planar conformation with a maximum deviation of 0.006 (1) Å for S1 in mol­ecule 1 and of 0.003 (1) Å for S1*B* in mol­ecule 2. The phthalazine ring systems are also essentially planar with maximum deviations from the best plane of the ten-membered ring systems of 0.003 (1) Å for N1 in mol­ecule 1 and 0.022 (1) Å for C8*B* in mol­ecule 2. The lengths of the N4—C9 and N4*B*—C9*B* bonds of 1.294 (2) and 1.296 (2) Å, respectively, are in agreement with that of an N=C*sp* ^2^ bond (1.282 ±0.060) Å found in the CSD (Version 5.39, update of August 2018; Groom *et al.*, 2016 ▸) for acyclic nitro­gen and carbon atoms in organic compounds. This confirms the condensation reaction between the two reagents. The hydrogen atoms H2 and H2*B* bonded respectively to N2 and N2*B* (see Table 1 ▸) indicate that proton transfer from the imino nitro­gen atoms N3 and N3*B* has occurred. The latter is confirmed by the double-bond character of N3—C8 [1.306 (2) Å] and N3*B*—C8*B* [1.309 (2) Å] and the single-bond character of N3—N4 [1.398 (2) Å] and N3*B*—N4*B* [1.400 (2) Å]. Indeed, these values are in agreement with the bond lengths for C=N and N-N bonds (1.3 ± 0.1 and 1.4 ± 0.1 Å, respectively) in the C=N—N fragment with a cyclic carbon atom and acyclic nitro­gen atoms for organic compounds in the CSD. Such a proton transfer has been reported in other hydrazinophthal­azine derivatives (Ianelli *et al.*, 2002 ▸; Butcher *et al.*, 2007 ▸; Popov *et al.*, 2012 ▸; Nfor *et al.*, 2013 ▸; Majoumo-Mbe *et al.*, 2015 ▸).

## Supra­molecular features   

**Figure 3:**
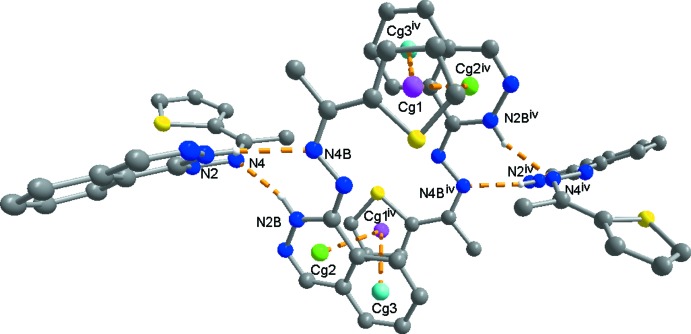
The packing of dimers of mol­ecules 1 and 2 (symmetry code as in Table 1 ▸).

**Figure 4:**
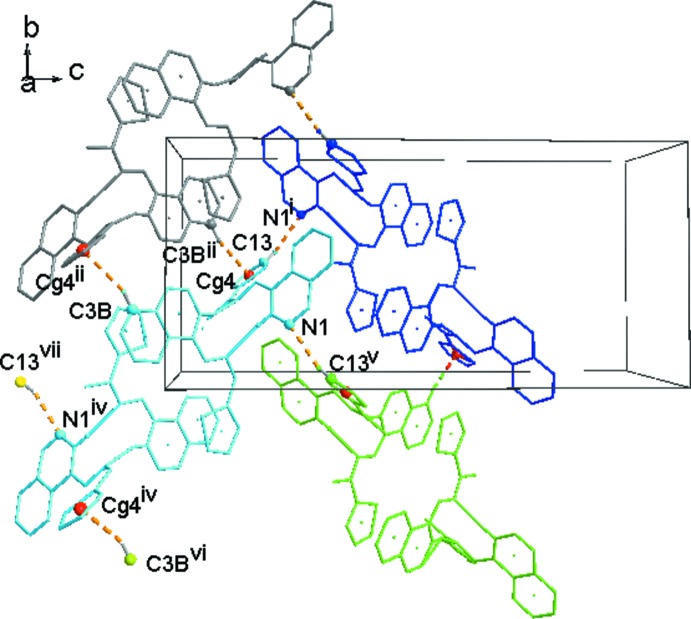
Packing mode of the tetra­mers in a herringbone motif. [Symmetry codes: (i) −*x* + 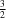, *y* + 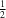, −*z* + 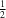; (ii) −*x* + 1, −*y* + 1, −*z*; (iv) −*x* + 1, −*y*, −*z*; (v) −*x* + 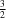, *y* − 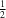, −*z* + 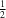; (vi) *x*, *y* − 1, *z*; (vii) *x* − 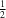, −*y* + 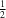, *z* − 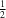].

**Figure 5:**
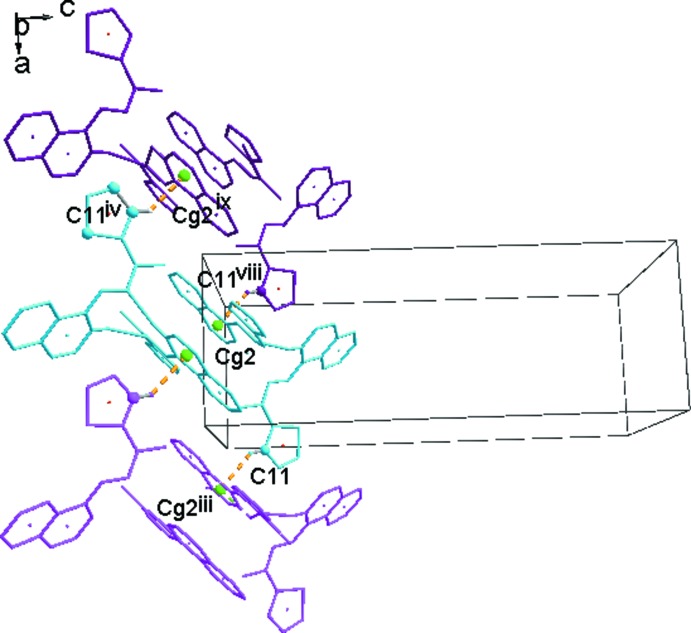
Packing mode of the tetra­mers in the *ac* plane. [Symmetry codes: (iii) 1 + *x*, *y*, *z*; (viii) *x* − 1, *y*, *z*; (ix) −*x*, −*y*, −*z*.]

Mol­ecules 1 and 2 are linked *via* two N—H⋯N hydrogen bonds (see Table 1 ▸), forming dimers which are held together by two bifurcated π–π inter­actions (Table 1 ▸) between the phthalazine and thio­phene moieties, as shown in Fig. 3 ▸. Similar bifurcated π–π inter­actions are also observed in 3-(benzo­thia­zol-2-yl)thio­phene (Nguyen Ngoc *et al.*, 2017 ▸). The resulting tetra­mers in the title compound are packed in a tilted herringbone motif. As shown in Fig. 4 ▸, they inter­act *via* the C13—H13⋯N1^i^ hydrogen bonds and C3*B*—H3*B*⋯*Cg*4^ii^ inter­actions along the *b*-axis direction (Fig. 4 ▸) and in the *ac* plane *via* C11—H11⋯*Cg*2^iii^ inter­actions (Fig. 5 ▸). The resulting packing shows small voids of 12.94 Å^3^ (0.5% of the unit cell; calculated with a probe radius of 1.2 Å by using the contact surface).

## Database survey   

A search of the Cambridge Structural Database (CSD, Version 5.39, update of August 2018; Groom *et al.*, 2016 ▸) for 2-acetyl­thio­phene-1-phthalazinylhydrazone derivatives did not give any hits. A search for structures in which the phthalazine ring exhibits bifurcated π–π inter­actions similar to those observed in the title structure gave six hits for organic compounds, all of which have six-membered rings: GUTYAX, GUTYOL, GUTYUR and GUTZIG (Trzesowska-Kruszynska, 2015 ▸), HILWAB (Büyükgüngör *et al.*, 2007 ▸) and TOMKIR (Bakale *et al.*, 2014*a* ▸). None of these crystals exhibits a packing mode with a tetra­meric motif similar to that reported in this work.

## Synthesis and crystallization   

The title mol­ecule was prepared by condensation of 2-acetyl­thio­phene (2.54 g, 20 mmol) and hydralazine hydro­chloride (3.94 g, 20 mmol) in 20 ml of methanol and 10 ml of aqueous solution of sodium acetate (1.64 g, 20 mmol) as buffering agent. The mixture was refluxed at 338 K under stirring for four h. The product was left overnight to cool. The yellow precipitate was filtered off and washed several times with water and methanol, and finally crystallized from a mixture of DMF/methanol (2:1) as yellow crystals (in a yield of around 80%) suitable for single-crystal X-ray diffraction studies.

## Refinement   

Crystal data, data collection and structure refinement details are summarized in Table 2 ▸. All H atoms could be located in difference-density Fourier maps. They were refined isotropically with *U*_iso_(H)= 1.2*U*_eq_(C,N).

## Supplementary Material

Crystal structure: contains datablock(s) I. DOI: 10.1107/S2056989019000732/lh5889sup1.cif

Supporting information file. DOI: 10.1107/S2056989019000732/lh5889Isup2.cml

CCDC reference: 1891271

Additional supporting information:  crystallographic information; 3D view; checkCIF report

## Figures and Tables

**Table 1:** Hydrogen-bond geometry (Å, °) *Cg*1–4 are the centroids of the S1*B*/C10*B*–C13*B*, N1*B*/C1*B*/C2*B*/C7*B*/C8*B*/N2*B*, C2*B*–C7*B*, and S1/C10–C13 rings, respectively.

*D*—H⋯*A*	*D*—H	H⋯*A*	*D*⋯*A*	*D*—H⋯*A*
N2—H2⋯N4*B*	0.90 (2)	2.26 (2)	3.131 (2)	165 (2)
C13—H13⋯N1^i^	0.94 (2)	2.61 (2)	3.387 (2)	140 (2)
N2*B*—H2*B*⋯N4	0.91 (2)	2.04 (2)	2.897 (2)	157 (2)
C3*B*—H3*B*⋯*Cg*4^ii^	0.96 (2)	2.94 (2)	3.808 (2)	151 (2)
C11—H11⋯*Cg*2^iii^	0.93 (2)	2.59 (2)	3.3796 (19)	143 (2)
*Cg*1⋯*Cg*2^iv^			3.519 (2)	
*Cg*1⋯*Cg*3^iv^			3.829 (2)	

Symmetry codes: (i) 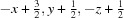; (ii) 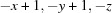; (iii) 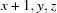; (iv) 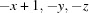.

**Table 2:** Experimental details

Crystal data	
Chemical formula	C_14_H_12_N_4_S
*M*_r_	268.34
Crystal system, space group	Monoclinic, *P*2_1_/*n*
Temperature (K)	130
*a*, *b*, *c* (Å)	8.9210 (2), 11.6792 (2), 24.7020 (4)
β (°)	90.051 (2)
*V* (Å^3^)	2573.70 (8)
*Z*	8
Radiation type	Mo *K*α
μ (mm^−1^)	0.24
Crystal size (mm)	0.2 × 0.2 × 0.1

Data collection	
Diffractometer	Agilent Xcalibur, Sapphire3, Gemini
Absorption correction	Multi-scan (*CrysAlis PRO*; Agilent, 2014 ▸)
*T*_min_, *T*_max_	0.995, 1
No. of measured, independent and observed [*I* > 2σ(*I*)] reflections	22778, 7849, 5743
*R*_int_	0.043
(sin θ/λ)_max_ (Å^−1^)	0.714

Refinement	
*R*[*F* ^2^ > 2σ(*F* ^2^)], *wR*(*F* ^2^), *S*	0.050, 0.105, 1.02
No. of reflections	7849
No. of parameters	415
H-atom treatment	Only H-atom coordinates refined
Δρ_max_, Δρ_min_ (e Å^−3^)	0.32, −0.40

Computer programs: *CrysAlis PRO* (Agilent, 2014 ▸), *SHELXS97* (Sheldrick, 2008 ▸), *SHELXL2018* (Sheldrick, 2015 ▸), *DIAMOND* (Brandenburg, 1999 ▸), *OLEX2* (Dolomanov *et al.*, 2009 ▸), *publCIF* (Westrip, 2010 ▸), *PLATON* (Spek, 2009 ▸) and *enCIFer* (Allen *et al.*, 2004 ▸).

## supplementary crystallographic information

### Crystal data

**Table:**

C_14_H_12_N_4_S	*F*(000) = 1120
*M**_r_* = 268.34	*D*_x_ = 1.385 Mg m^−^^3^
Monoclinic, *P*2_1_/*n*	Mo *K*α radiation, λ = 0.71073 Å
*a* = 8.9210 (2) Å	Cell parameters from 5823 reflections
*b* = 11.6792 (2) Å	θ = 2.9–31.2°
*c* = 24.7020 (4) Å	µ = 0.24 mm^−^^1^
β = 90.051 (2)°	*T* = 130 K
*V* = 2573.70 (8) Å^3^	Prism, clear intense yellow
*Z* = 8	0.2 × 0.2 × 0.1 mm

### Data collection

**Table:**

Agilent Xcalibur, Sapphire3, Gemini diffractometer	7849 independent reflections
Radiation source: Enhance (Mo) X-ray Source	5743 reflections with *I* > 2σ(*I*)
Detector resolution: 16.356 pixels mm^-1^	*R*_int_ = 0.043
ω scans	θ_max_ = 30.5°, θ_min_ = 2.4°
Absorption correction: multi-scan (CrysAlis PRO; Agilent, 2014)	*h* = −12→12
*T*_min_ = 0.995, *T*_max_ = 1	*k* = −16→14
22778 measured reflections	*l* = −34→35

### Refinement

**Table:**

Refinement on *F*^2^	Primary atom site location: structure-invariant direct methods
Least-squares matrix: full	Secondary atom site location: difference Fourier map
*R*[*F*^2^ > 2σ(*F*^2^)] = 0.050	Hydrogen site location: difference Fourier map
*wR*(*F*^2^) = 0.105	Only H-atom coordinates refined
*S* = 1.02	*w* = 1/[σ^2^(*F*_o_^2^) + (0.0334*P*)^2^ + 1.0293*P*] where *P* = (*F*_o_^2^ + 2*F*_c_^2^)/3
7849 reflections	(Δ/σ)_max_ < 0.001
415 parameters	Δρ_max_ = 0.32 e Å^−^^3^
0 restraints	Δρ_min_ = −0.40 e Å^−^^3^

### Special details

**Table:**

Geometry. All esds (except the esd in the dihedral angle between two l.s. planes) are estimated using the full covariance matrix. The cell esds are taken into account individually in the estimation of esds in distances, angles and torsion angles; correlations between esds in cell parameters are only used when they are defined by crystal symmetry. An approximate (isotropic) treatment of cell esds is used for estimating esds involving l.s. planes.

### Fractional atomic coordinates and isotropic or equivalent isotropic displacement parameters (Å^2^)

**Table:**

	*x*	*y*	*z*	*U*_iso_*/*U*_eq_	
S1	0.94607 (5)	0.48983 (4)	0.18553 (2)	0.02313 (10)	
N1	0.38700 (18)	0.24390 (13)	0.23474 (6)	0.0318 (4)	
N2	0.49212 (17)	0.28369 (13)	0.19925 (6)	0.0262 (3)	
N3	0.66086 (16)	0.42226 (12)	0.16566 (6)	0.0230 (3)	
N4	0.68717 (16)	0.34078 (12)	0.12535 (6)	0.0242 (3)	
C1	0.3446 (2)	0.31335 (17)	0.27251 (8)	0.0308 (4)	
C2	0.39880 (19)	0.42891 (15)	0.27863 (7)	0.0235 (3)	
C3	0.3449 (2)	0.50298 (17)	0.31884 (7)	0.0294 (4)	
C4	0.3953 (2)	0.61410 (17)	0.32093 (7)	0.0307 (4)	
C5	0.5009 (2)	0.65325 (16)	0.28398 (8)	0.0296 (4)	
C6	0.5571 (2)	0.58127 (15)	0.24471 (7)	0.0232 (3)	
C7	0.50579 (18)	0.46846 (14)	0.24163 (6)	0.0199 (3)	
C8	0.55718 (18)	0.38990 (14)	0.19964 (6)	0.0207 (3)	
C9	0.82401 (19)	0.33588 (14)	0.10820 (6)	0.0218 (3)	
C10	0.95080 (18)	0.39951 (14)	0.12963 (6)	0.0204 (3)	
C11	1.0950 (2)	0.39353 (15)	0.10989 (7)	0.0256 (4)	
C12	1.1994 (2)	0.45958 (16)	0.13921 (8)	0.0300 (4)	
C13	1.1348 (2)	0.51514 (16)	0.18139 (8)	0.0296 (4)	
C14	0.8531 (2)	0.25334 (18)	0.06250 (8)	0.0307 (4)	
H2	0.512 (2)	0.2355 (18)	0.1720 (8)	0.037*	
H1	0.269 (2)	0.2820 (17)	0.2976 (8)	0.037*	
H3	0.274 (2)	0.4743 (17)	0.3430 (8)	0.037*	
H4	0.358 (2)	0.6664 (18)	0.3478 (8)	0.037*	
H5	0.535 (2)	0.7318 (18)	0.2848 (8)	0.037*	
H6	0.631 (2)	0.6036 (17)	0.2200 (8)	0.037*	
H11	1.119 (2)	0.3497 (18)	0.0797 (9)	0.037*	
H12	1.304 (2)	0.4642 (17)	0.1304 (8)	0.037*	
H13	1.178 (2)	0.5639 (18)	0.2072 (8)	0.037*	
H14A	0.893 (2)	0.2920 (18)	0.0310 (9)	0.037*	
H14B	0.932 (2)	0.1956 (18)	0.0732 (8)	0.037*	
H14C	0.764 (2)	0.2144 (18)	0.0526 (8)	0.037*	
S1B	0.36400 (5)	−0.14245 (4)	0.06118 (2)	0.02429 (10)	
N1B	0.41957 (17)	0.39056 (12)	0.03070 (6)	0.0272 (3)	
N2B	0.43791 (16)	0.28603 (12)	0.05466 (6)	0.0227 (3)	
N3B	0.38510 (15)	0.08995 (12)	0.06759 (6)	0.0224 (3)	
N4B	0.49714 (16)	0.09992 (12)	0.10701 (6)	0.0232 (3)	
C1B	0.3204 (2)	0.39742 (16)	−0.00702 (7)	0.0278 (4)	
C2B	0.22946 (19)	0.30252 (15)	−0.02516 (7)	0.0241 (3)	
C3B	0.1233 (2)	0.31324 (17)	−0.06684 (7)	0.0308 (4)	
C4B	0.0420 (2)	0.21961 (18)	−0.08347 (7)	0.0310 (4)	
C5B	0.0654 (2)	0.11288 (16)	−0.05935 (7)	0.0280 (4)	
C6B	0.16841 (19)	0.10032 (15)	−0.01843 (7)	0.0237 (3)	
C7B	0.25136 (18)	0.19541 (14)	−0.00046 (6)	0.0208 (3)	
C8B	0.36158 (18)	0.18738 (14)	0.04280 (6)	0.0203 (3)	
C9B	0.54790 (18)	0.00318 (14)	0.12500 (6)	0.0215 (3)	
C10B	0.49778 (18)	−0.11113 (14)	0.10992 (6)	0.0213 (3)	
C11B	0.5481 (2)	−0.21069 (16)	0.13366 (7)	0.0273 (4)	
C12B	0.4792 (2)	−0.31014 (17)	0.11303 (8)	0.0321 (4)	
C13B	0.3777 (2)	−0.28606 (16)	0.07399 (8)	0.0302 (4)	
C14B	0.6704 (2)	0.01133 (18)	0.16641 (8)	0.0287 (4)	
H2B	0.512 (2)	0.2829 (17)	0.0797 (8)	0.034*	
H1B	0.310 (2)	0.4713 (18)	−0.0238 (8)	0.034*	
H3B	0.110 (2)	0.3875 (18)	−0.0833 (8)	0.034*	
H4B	−0.029 (2)	0.2277 (17)	−0.1119 (8)	0.034*	
H5B	0.008 (2)	0.0479 (18)	−0.0722 (8)	0.034*	
H6B	0.184 (2)	0.0280 (18)	−0.0021 (8)	0.034*	
H11B	0.623 (2)	−0.2118 (17)	0.1613 (8)	0.034*	
H12B	0.499 (2)	−0.3842 (18)	0.1251 (8)	0.034*	
H13B	0.318 (2)	−0.3381 (18)	0.0541 (8)	0.034*	
H14D	0.639 (2)	−0.0227 (17)	0.2010 (9)	0.034*	
H14E	0.696 (2)	0.0877 (18)	0.1720 (8)	0.034*	
H14F	0.759 (2)	−0.0329 (17)	0.1540 (8)	0.034*	

### Atomic displacement parameters (Å^2^)

**Table:**

	*U*^11^	*U*^22^	*U*^33^	*U*^12^	*U*^13^	*U*^23^
S1	0.0273 (2)	0.0221 (2)	0.02000 (19)	−0.00066 (16)	−0.00166 (16)	−0.00281 (16)
N1	0.0351 (9)	0.0281 (8)	0.0324 (8)	−0.0089 (7)	0.0047 (7)	0.0011 (7)
N2	0.0319 (8)	0.0216 (7)	0.0250 (7)	−0.0041 (6)	0.0033 (6)	−0.0033 (6)
N3	0.0234 (7)	0.0238 (7)	0.0218 (7)	−0.0014 (6)	0.0006 (6)	−0.0056 (6)
N4	0.0254 (7)	0.0253 (7)	0.0219 (7)	−0.0024 (6)	0.0013 (6)	−0.0062 (6)
C1	0.0314 (10)	0.0323 (10)	0.0287 (9)	−0.0063 (8)	0.0050 (8)	0.0020 (8)
C2	0.0228 (8)	0.0265 (9)	0.0213 (8)	0.0015 (7)	−0.0024 (6)	0.0012 (7)
C3	0.0257 (9)	0.0384 (11)	0.0240 (9)	0.0051 (8)	0.0019 (7)	−0.0001 (8)
C4	0.0331 (10)	0.0342 (10)	0.0248 (9)	0.0101 (8)	−0.0035 (7)	−0.0082 (8)
C5	0.0352 (10)	0.0243 (9)	0.0293 (9)	0.0034 (8)	−0.0065 (8)	−0.0047 (8)
C6	0.0247 (8)	0.0227 (8)	0.0222 (8)	0.0002 (7)	−0.0037 (7)	0.0005 (7)
C7	0.0190 (8)	0.0229 (8)	0.0178 (7)	0.0018 (6)	−0.0038 (6)	−0.0014 (6)
C8	0.0199 (8)	0.0207 (8)	0.0214 (8)	0.0009 (6)	−0.0034 (6)	−0.0016 (6)
C9	0.0247 (8)	0.0214 (8)	0.0192 (7)	−0.0015 (6)	−0.0004 (6)	−0.0011 (6)
C10	0.0255 (8)	0.0176 (7)	0.0181 (7)	0.0008 (6)	−0.0010 (6)	0.0000 (6)
C11	0.0249 (9)	0.0249 (9)	0.0270 (9)	−0.0012 (7)	0.0024 (7)	−0.0024 (7)
C12	0.0230 (9)	0.0290 (10)	0.0380 (10)	−0.0028 (7)	−0.0013 (8)	−0.0039 (8)
C13	0.0304 (9)	0.0264 (9)	0.0319 (10)	−0.0041 (7)	−0.0070 (8)	−0.0030 (8)
C14	0.0283 (10)	0.0369 (11)	0.0271 (9)	−0.0037 (8)	0.0014 (8)	−0.0114 (8)
S1B	0.0258 (2)	0.0226 (2)	0.0244 (2)	−0.00187 (17)	−0.00405 (16)	−0.00243 (17)
N1B	0.0334 (8)	0.0215 (7)	0.0267 (7)	−0.0059 (6)	0.0031 (6)	0.0010 (6)
N2B	0.0247 (7)	0.0217 (7)	0.0217 (7)	−0.0039 (6)	−0.0014 (6)	0.0000 (6)
N3B	0.0229 (7)	0.0221 (7)	0.0223 (7)	−0.0035 (6)	−0.0045 (6)	−0.0007 (6)
N4B	0.0234 (7)	0.0246 (7)	0.0218 (7)	−0.0022 (6)	−0.0051 (6)	−0.0020 (6)
C1B	0.0344 (10)	0.0211 (8)	0.0280 (9)	−0.0034 (7)	0.0011 (8)	0.0033 (7)
C2B	0.0263 (9)	0.0243 (8)	0.0216 (8)	−0.0011 (7)	0.0021 (7)	0.0025 (7)
C3B	0.0360 (10)	0.0305 (10)	0.0260 (9)	0.0019 (8)	−0.0032 (8)	0.0075 (8)
C4B	0.0279 (9)	0.0410 (11)	0.0240 (9)	−0.0005 (8)	−0.0056 (7)	0.0032 (8)
C5B	0.0270 (9)	0.0307 (10)	0.0263 (9)	−0.0053 (7)	−0.0023 (7)	−0.0012 (8)
C6B	0.0239 (8)	0.0226 (8)	0.0245 (8)	−0.0030 (7)	0.0007 (7)	0.0002 (7)
C7B	0.0199 (8)	0.0233 (8)	0.0192 (7)	−0.0019 (6)	0.0026 (6)	−0.0013 (6)
C8B	0.0205 (8)	0.0213 (8)	0.0191 (7)	−0.0022 (6)	0.0026 (6)	−0.0016 (6)
C9B	0.0220 (8)	0.0252 (8)	0.0173 (7)	−0.0022 (6)	−0.0006 (6)	−0.0017 (6)
C10B	0.0208 (8)	0.0242 (8)	0.0190 (7)	−0.0014 (6)	0.0007 (6)	−0.0012 (6)
C11B	0.0287 (9)	0.0261 (9)	0.0271 (9)	−0.0009 (7)	−0.0034 (7)	0.0028 (7)
C12B	0.0365 (11)	0.0230 (9)	0.0369 (10)	−0.0028 (8)	−0.0015 (8)	0.0037 (8)
C13B	0.0345 (10)	0.0223 (9)	0.0337 (10)	−0.0049 (8)	−0.0016 (8)	−0.0045 (8)
C14B	0.0302 (10)	0.0305 (10)	0.0255 (9)	0.0001 (8)	−0.0082 (7)	−0.0022 (8)

### Geometric parameters (Å, º)

**Table:**

S1—C10	1.7383 (16)	S1B—C10B	1.7336 (17)
S1—C13	1.7122 (19)	S1B—C13B	1.7112 (19)
N1—N2	1.366 (2)	N1B—N2B	1.366 (2)
N1—C1	1.293 (2)	N1B—C1B	1.287 (2)
N2—C8	1.369 (2)	N2B—C8B	1.370 (2)
N2—H2	0.90 (2)	N2B—H2B	0.91 (2)
N3—N4	1.3975 (18)	N3B—N4B	1.3996 (19)
N3—C8	1.306 (2)	N3B—C8B	1.309 (2)
N4—C9	1.294 (2)	N4B—C9B	1.296 (2)
C1—C2	1.442 (3)	C1B—C2B	1.445 (2)
C1—H1	0.99 (2)	C1B—H1B	0.96 (2)
C2—C3	1.402 (2)	C2B—C3B	1.404 (2)
C2—C7	1.400 (2)	C2B—C7B	1.405 (2)
C3—C4	1.374 (3)	C3B—C4B	1.375 (3)
C3—H3	0.93 (2)	C3B—H3B	0.96 (2)
C4—C5	1.389 (3)	C4B—C5B	1.397 (3)
C4—H4	0.96 (2)	C4B—H4B	0.95 (2)
C5—C6	1.379 (2)	C5B—C6B	1.373 (2)
C5—H5	0.97 (2)	C5B—H5B	0.97 (2)
C6—C7	1.397 (2)	C6B—C7B	1.406 (2)
C6—H6	0.93 (2)	C6B—H6B	0.95 (2)
C7—C8	1.459 (2)	C7B—C8B	1.454 (2)
C9—C10	1.453 (2)	C9B—C10B	1.456 (2)
C9—C14	1.507 (2)	C9B—C14B	1.499 (2)
C10—C11	1.378 (2)	C10B—C11B	1.377 (2)
C11—C12	1.409 (3)	C11B—C12B	1.409 (3)
C11—H11	0.93 (2)	C11B—H11B	0.96 (2)
C12—C13	1.357 (3)	C12B—C13B	1.352 (3)
C12—H12	0.96 (2)	C12B—H12B	0.93 (2)
C13—H13	0.94 (2)	C13B—H13B	0.94 (2)
C14—H14A	0.97 (2)	C14B—H14D	0.98 (2)
C14—H14B	1.01 (2)	C14B—H14E	0.93 (2)
C14—H14C	0.95 (2)	C14B—H14F	0.99 (2)
			
C13—S1—C10	91.87 (9)	C13B—S1B—C10B	91.68 (9)
C1—N1—N2	116.82 (15)	C1B—N1B—N2B	116.82 (15)
N1—N2—C8	126.50 (15)	N1B—N2B—C8B	126.83 (15)
N1—N2—H2	113.9 (13)	N1B—N2B—H2B	114.7 (13)
C8—N2—H2	119.4 (13)	C8B—N2B—H2B	118.4 (13)
C8—N3—N4	112.37 (14)	C8B—N3B—N4B	111.54 (13)
C9—N4—N3	115.00 (14)	C9B—N4B—N3B	114.53 (14)
N1—C1—C2	124.37 (17)	N1B—C1B—C2B	124.21 (17)
N1—C1—H1	114.9 (12)	N1B—C1B—H1B	115.9 (12)
C2—C1—H1	120.7 (12)	C2B—C1B—H1B	119.9 (12)
C3—C2—C1	122.45 (16)	C3B—C2B—C1B	122.50 (16)
C7—C2—C1	117.98 (16)	C3B—C2B—C7B	119.42 (16)
C7—C2—C3	119.54 (16)	C7B—C2B—C1B	118.07 (16)
C2—C3—H3	117.7 (13)	C2B—C3B—H3B	118.1 (12)
C4—C3—C2	119.78 (17)	C4B—C3B—C2B	120.23 (17)
C4—C3—H3	122.5 (13)	C4B—C3B—H3B	121.7 (12)
C3—C4—C5	120.53 (17)	C3B—C4B—C5B	120.24 (17)
C3—C4—H4	120.8 (13)	C3B—C4B—H4B	119.5 (12)
C5—C4—H4	118.7 (13)	C5B—C4B—H4B	120.2 (12)
C4—C5—H5	120.9 (12)	C4B—C5B—H5B	118.6 (12)
C6—C5—C4	120.59 (18)	C6B—C5B—C4B	120.59 (17)
C6—C5—H5	118.5 (12)	C6B—C5B—H5B	120.8 (12)
C5—C6—C7	119.60 (17)	C5B—C6B—C7B	119.97 (17)
C5—C6—H6	123.1 (13)	C5B—C6B—H6B	120.4 (13)
C7—C6—H6	117.3 (13)	C7B—C6B—H6B	119.6 (13)
C2—C7—C8	118.12 (15)	C2B—C7B—C6B	119.54 (16)
C6—C7—C2	119.94 (15)	C2B—C7B—C8B	118.03 (15)
C6—C7—C8	121.92 (15)	C6B—C7B—C8B	122.42 (15)
N2—C8—C7	116.19 (14)	N2B—C8B—C7B	116.01 (15)
N3—C8—N2	123.93 (15)	N3B—C8B—N2B	123.47 (15)
N3—C8—C7	119.88 (15)	N3B—C8B—C7B	120.52 (15)
N4—C9—C10	126.35 (15)	N4B—C9B—C10B	127.20 (15)
N4—C9—C14	115.90 (15)	N4B—C9B—C14B	115.65 (15)
C10—C9—C14	117.73 (15)	C10B—C9B—C14B	117.14 (15)
C9—C10—S1	125.48 (12)	C9B—C10B—S1B	125.60 (13)
C11—C10—S1	109.58 (13)	C11B—C10B—S1B	109.97 (13)
C11—C10—C9	124.89 (15)	C11B—C10B—C9B	124.41 (16)
C10—C11—C12	114.04 (16)	C10B—C11B—C12B	113.57 (17)
C10—C11—H11	121.9 (13)	C10B—C11B—H11B	122.9 (12)
C12—C11—H11	124.1 (13)	C12B—C11B—H11B	123.5 (12)
C11—C12—H12	123.9 (12)	C11B—C12B—H12B	124.4 (13)
C13—C12—C11	112.03 (17)	C13B—C12B—C11B	112.22 (17)
C13—C12—H12	124.1 (12)	C13B—C12B—H12B	123.4 (13)
S1—C13—H13	118.1 (13)	S1B—C13B—H13B	119.7 (13)
C12—C13—S1	112.47 (14)	C12B—C13B—S1B	112.55 (15)
C12—C13—H13	129.4 (13)	C12B—C13B—H13B	127.8 (13)
C9—C14—H14A	111.6 (12)	C9B—C14B—H14D	111.0 (12)
C9—C14—H14B	110.6 (12)	C9B—C14B—H14E	110.1 (13)
C9—C14—H14C	110.8 (13)	C9B—C14B—H14F	109.7 (12)
H14A—C14—H14B	105.4 (17)	H14D—C14B—H14E	109.2 (17)
H14A—C14—H14C	108.9 (18)	H14D—C14B—H14F	106.6 (16)
H14B—C14—H14C	109.3 (17)	H14E—C14B—H14F	110.1 (17)

### Hydrogen-bond geometry (Å, º)

*Cg*1–4 are the centroids of the S1*B*/C10*B*–C13*B*, N1*B*/C1*B*/C2*B*/C7*B*/C8*B*/N2*B*, C2*B*–C7*B*, and S1/C10–C13 rings, respectively.

**Table:**

*D*—H···*A*	*D*—H	H···*A*	*D*···*A*	*D*—H···*A*
N2—H2···N4*B*	0.90 (2)	2.26 (2)	3.131 (2)	165 (2)
C13—H13···N1^i^	0.94 (2)	2.61 (2)	3.387 (2)	140 (2)
N2*B*—H2*B*···N4	0.91 (2)	2.04 (2)	2.897 (2)	157 (2)
C3*B*—H3*B*···*Cg*4^ii^	0.96 (2)	2.94 (2)	3.808 (2)	151 (2)
C11—H11···*Cg*2^iii^	0.93 (2)	2.59 (2)	3.3796 (19)	143 (2)
*Cg*1···*Cg*2^iv^			3.519 (2)	
*Cg*1···*Cg*3^iv^			3.829 (2)	

Symmetry codes: (i) −*x*+3/2, *y*+1/2, −*z*+1/2; (ii) −*x*+1, −*y*+1, −*z*; (iii) *x*+1, *y*, *z*; (iv) −*x*+1, −*y*, −*z*.
